# Dynamics and Control of Flagella Assembly in *Salmonella typhimurium*

**DOI:** 10.3389/fcimb.2018.00036

**Published:** 2018-02-08

**Authors:** Chandrani Das, Chaitanya Mokashi, Sharmila S. Mande, Supreet Saini

**Affiliations:** ^1^Department of Chemical Engineering, Indian Institute of Technology Bombay, Mumbai, India; ^2^Bio-Sciences R&D Division, TCS Research, Tata Consultancy Services Limited, Pune, India

**Keywords:** *Salmonella typhimurium*, flagella, mathematical model, gene regulation, gene expression dynamics

## Abstract

The food-borne pathogen *Salmonella typhimurium* is a common cause of infections and diseases in a wide range of hosts. One of the major virulence factors associated to the infection process is flagella, which helps the bacterium swim to its preferred site of infection inside the host, the M-cells (Microfold cells) lining the lumen of the small intestine. The expression of flagellar genes is controlled by an intricate regulatory network. In this work, we investigate two aspects of flagella regulation and assembly: (a) distribution of the number of flagella in an isogenic population of bacteria and (b) dynamics of gene expression post cell division. More precisely, in a population of bacteria, we note a normal distribution of number of flagella assembled per cell. How is this distribution controlled, and what are the key regulators in the network which help the cell achieve this? In the second question, we explore the role of protein secretion in dictating gene expression dynamics post cell-division (when the number of hook basal bodies on the cell surface is reduced by a factor of two). We develop a mathematical model and perform stochastic simulations to address these questions. Simulations of the model predict that two accessory regulators of flagella gene expression, FliZ and FliT, have significant roles in maintaining population level distribution of flagella. In addition, FliT and FlgM were predicted to control the level and temporal order of flagellar gene expression when the cell adapts to post cell division consequences. Further, the model predicts that, the FliZ and FliT dependent feedback loops function under certain thresholds, alterations in which can substantially affect kinetics of flagellar genes. Thus, based on our results we propose that, the proteins FlgM, FliZ, and FliT, thought to have accessory roles in regulation of flagella, likely play a critical role controlling gene expression during cell division, and frequency distribution of flagella.

## Introduction

*Salmonella enterica* serovar typhimurium (*S. typhimurium*) is a food-borne pathogen associated with a number of diseases in a wide range of hosts. Depending on the host and the particular strain of the bacterium, infections can be localized to the gastrointestinal tract and local lymphatics, or spread to organs like spleen, liver, etc. (Everest et al., 1999). Severe cases of these infections/diseases may lead to several clinical complications including death (Zhang et al., 2003). Although overall burden of food borne infections has been reduced in the last few decades, the incidence of *S. typhimurium* related illness remained nearly consistent, indicating requirement of improved therapeutic regimes (DiMarzio et al., 2013). Moreover, emergence of multi-drug resistance strains and ability to spread the resistance factors to other strains are making the existing treatment strategies less effective.

One of the virulence factors involved in the initial phase of the infection process is the flagella. Flagella are long helical, rotatable appendages which are located on the bacterial cell surface, and enable the bacterium to swim in liquid media and swarm upon solid surfaces. Upon infection, it is thought that the flagella help the bacterium reach the invasion site, Microfold (M)-cells, lining the small intestine (Jones et al., 1992; Saini et al., 2011).

The structural components of flagella mainly include three elements—basal body, hook, and filament (Macnab, 1999). While the basal body acts as an anchor to the flagellum by attaching it to the cell membrane, rotation of the helical filament drives forward movement. The hook serves as a flexible linker between the basal body and the rigid filament. Additionally, chemotaxis and motor proteins help the bacterium sense the environment and move in an appropriate direction. Around 50 genes, arranged in more than 17 operons, which include structural components of the flagella, regulators, chaperones, are known to be involved in the assembly of flagella (Chilcott and Hughes, 2000). Moreover, these genes have been shown to be expressed in hierarchical manner consistent with the assembly process (Kalir et al., 2001).

**Figure 1:**
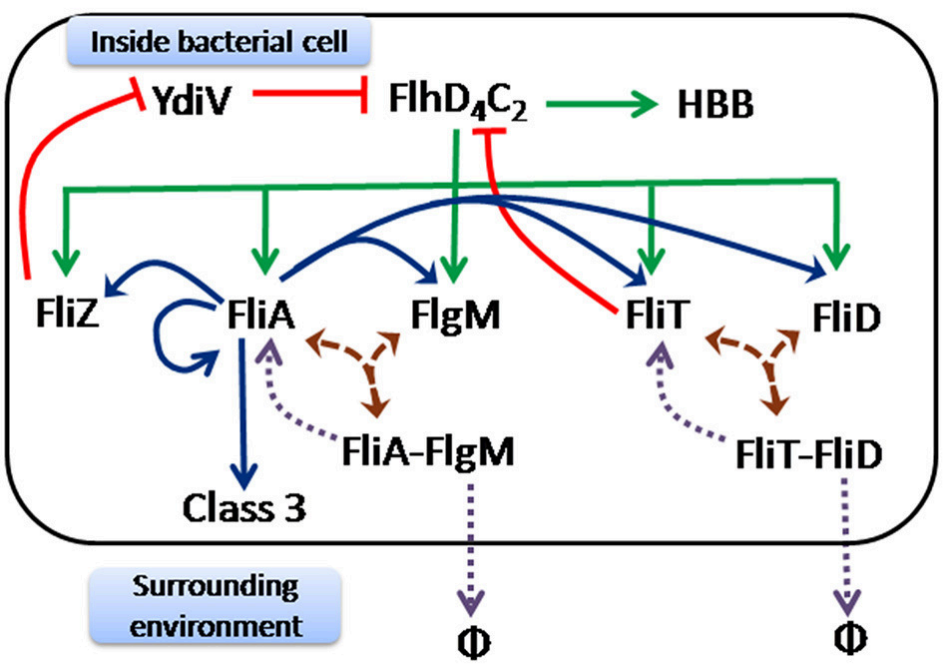
Regulatory network controlling expression of flagellar genes in *S. typhimurium*. The master regulator of flagella FlhD_4_C_2_ is at the top hierarchy of the regulatory cascade. Once it is expressed upon receiving multiple internal and external signals, a series of regulatory events take place for timely and proper expression of flagellar genes. The initial events have been represented by green arrows. These include activation of class 2 genes encoding HBB (hook, basal body) by FlhD_4_C_2_. At the same time, FlhD_4_C_2_ also activates the genes encoding five regulators—FliA (σ factor), FlgM (anti- σ factor), FliD (cap protein), FliT (anti- FlhD_4_C_2_ factor), and FliZ (regulator). The subsequent events have been depicted by brown arrows which include post-translational regulation through formation of two protein complexes FliA-FlgM (complex of FliA and FlgM) and FliT-FliD (complex of FliD and FliT). These complexes get disassociated only after complete assembly of HBB. The subsequent events after disassociation of these two protein complexes have been shown by purple dotted arrows. As the protein complexes get disrupted, FlgM and FliD are exported out of the cell (represented by φ), leaving free FliA and FliT inside the cell. Consequently, FliA carries out the next set of events represented by blue arrows. FliA activates class 3 genes encoding filament, motor and chemotaxis proteins. At the same time, FliA also activates itself and other class 2 genes encoding FliZ, FlgM, FliD and FliT. Finally, two feedback loops, represented by red edges, act on the master regulator FlhD_4_C_2_. These include FliZ dependent positive and FliT dependent negative feedback loops. FliZ acts as a repressor of a non-flagellar protein YdiV, which participates in nutritional status (of the cell) dependent regulation of FlhD_4_C_2_. FliT forms protein complex with FlhD_4_C_2_ and prevents FlhD_4_C_2_-dependent activation of class two genes.

The major regulators of the flagellar assembly include FlhD, FlhC, FliA, FliZ, FlgM, FliD, and FliT (Ohnishi et al., 1992; Liu and Matsumura, 1995; Yanagihara et al., 1999; Prüss et al., 2001; Aldridge et al., 2006, 2010; Yamamoto and Kutsukake, 2006; Saini et al., 2008; Figure 1). The promoters controlling expression of flagellar genes have been divided in three classes based on the timing of expression of the genes under their control (Chilcott and Hughes, 2000). A single class 1 promoter (P*flhDC* promoter) encodes two proteins, FlhD and FlhC, which come together to form a transcriptional activator complex FlhD_4_C_2_ (Kutsukake, 1997; Yanagihara et al., 1999; Chilcott and Hughes, 2000; Prüss et al., 2001). This promoter integrates inputs from a large number of global regulators, and makes the decision whether the cell will be motile or non-motile (Yanagihara et al., 1999; Ko and Park, 2000; Wei et al., 2001; Clegg and Hughes, 2002; Lehnen et al., 2002; Sperandio et al., 2002; Tomoyasu et al., 2002; Ellermeier and Slauch, 2003; Francez-Charlot et al., 2003; Teplitski et al., 2003; Clarke and Sperandio, 2005). The FlhD_4_C_2_ transcription factor is essential for activation of class 2 promoters (Liu and Matsumura, 1994; Liu et al., 1995; Ikebe et al., 1999a).

Class 2 promoters control expression of all proteins constituting the structural elements of the hook-basal body (HBB), filament cap protein (FliD), and regulators (FliA, FlgM, FliZ, and FliT) (Jones and Macnab, 1990; Ohnishi et al., 1990; Gillen and Hughes, 1993; Kutsukake and Ide, 1995; Yokoseki et al., 1995; Ikebe et al., 1999a). FliA is a flagella-specific sigma factor (σ^28^) and is responsible for activation of all class 3 promoters (Ohnishi et al., 1992; Liu and Matsumura, 1995), which control expression of proteins forming the filament and motor, and chemotaxis proteins (Ohnishi et al., 1992; Liu and Matsumura, 1995). In addition, FliA positively regulates its own expression, as well as that of the genes encoding FlgM, FliZ, FliD, and FliT (Gillen and Hughes, 1993; Kutsukake and Ide, 1995; Ikebe et al., 1999a,b; Kutsukake et al., 1999). Thus, the genes encoding these five proteins are under control of both class 2 and class 3 promoters.

In addition to direct control of transcription, several post-translational regulatory interactions play an important role in regulating the flagellar assembly process. First, FlgM, an anti-sigma factor for FliA, binds to FliA resulting in the FliA-FlgM protein complex (Ohnishi et al., 1992; Chadsey and Hughes, 2001; Aldridge et al., 2006). This sequestration of FliA by FlgM does not leave any free FliA in the cell to activate class 3 promoters (Chadsey et al., 1998; Chadsey and Hughes, 2001). Upon completion of a functional HBB, however, the FliA-FlgM complex interacts with the flagellar export apparatus resulting in secretion of FlgM out of the cell (Hughes et al., 1993; Kutsukake, 1994; Aldridge et al., 2006). Secretion of FlgM frees FliA to activate class 3 promoter (Hughes et al., 1993; Aldridge et al., 2006). The second interaction involves the cap protein, FliD and its chaperone, FliT (Fraser et al., 1999; Aldridge et al., 2010). FliD is secreted from the HBB and assembles at the tip of the flagella (Fraser et al., 1999; Bennett et al., 2001). Prior to completion of HBB, FliT binds FliD and forms a FliD-FliT complex inside the cell (Fraser et al., 1999; Bennett et al., 2001; Aldridge et al., 2010). On completion of the HBB, FliD is secreted through the HBB structure (Aldridge et al., 2010). The free FliT inside the cell interacts with the FlhD_4_C_2_ complex and prevents FlhD_4_C_2_-dependent activation of class 2 genes (Yamamoto and Kutsukake, 2006; Aldridge et al., 2010; Imada et al., 2010). This feedback loop is thought to negatively control expression of flagella genes in response to increased secretion of FliD from the cell (Yamamoto and Kutsukake, 2006).

In addition, FliS, expressed in the same operon as *fliD* and *fliT*, is known to be a chaperone for the filament protein, FliC. The absence of *fliS* is known to lead to production of shorter filament lengths in the flagella (Auvray et al., 2001; Galeva et al., 2014). Lastly, FlgN (expressed in an operon with the anti-sigma factor, FlgM) is known to be a chaperone for the hook-associated proteins (HAPs) FlgK and FlgL. The proteins FlgK and FlgL form the junction between the flexible hook and the rigid filament (Fraser et al., 1999).

An indirect positive feedback loop involving FliZ tunes flagellar genes expression (Wada et al., 2011b). FliZ negatively regulates the expression of YdiV by repressing expression from the P*ydiV* promoter (Wada et al., 2011b). YdiV acts via sequestering the FlhD_4_C_2_ complex and preventing the FlhD_4_C_2_-dependent activation of class 2 promoters (Wada et al., 2011b). In addition, YdiV binding to FlhD_4_C_2_ also enhances the FlhD_4_C_2_ degradation rates via ClpXP (Takaya et al., 2012). Physiologically, YdiV is known to be a sensor of the nutritional status of the cell, and therefore, it is thought that YdiV is the link between flagellar biosynthesis and metabolism in *Salmonella*.

A large number of experimental studies have led to a detailed understanding of the regulatory network controlling flagellar assembly in *Salmonella*. In addition, a few modeling efforts to analyze and understand the regulation of flagella assembly have also been reported recently (Saini et al., 2011; Jain et al., 2015). In this study we focus on two aspects associated with flagellar assembly dynamics. We develop a model describing the assembly process, and through stochastic simulations of the model, address the following questions.

- A wild type population of *Salmonella* is known to exhibit a normal distribution of flagella per cell. What or which regulatory interactions accounts for this variation in an isogenic population?
- *Salmonella* class 1 promoter (P*flhDC* promoter) is always in a fully or partially ON state (there are no experimental conditions where the promoter has been found to be in a completely OFF state). Since models analyzing flagellar assembly have focused on the gene expression dynamics as cells transit from a non-flagellated to a flagellated state, we do not yet understand how gene expression dynamics gets tuned as cells move from one flagellated state to another. Particularly, how do cells tune gene expression post cell-division?

Through our work, we explain and quantify the above factors in flagellar assembly in *Salmonella*.

## Materials and methods

### Mathematical model

The following regulatory network is represented in the mathematical model studied in this work. The FlhD_4_C_2_ complex acts as master regulator necessary for class 2 gene expression (Liu and Matsumura, 1994; Liu et al., 1995; Ikebe et al., 1999a) and the sigma factor FliA is essential for class 3 gene expression (Ohnishi et al., 1992; Liu and Matsumura, 1995). FlhD_4_C_2_ activates all class 2 genes i.e., HBB, FliA, FlgM, FliZ, FliT, and FliD (Liu and Matsumura, 1994; Liu et al., 1995; Ikebe et al., 1999a). FliA positively auto regulates itself as well as activates all class 3 genes, which include the filament, motor, and the chemotaxis proteins (Gillen and Hughes, 1993; Kutsukake and Ide, 1995; Ikebe et al., 1999a,b; Kutsukake et al., 1999). FliA and FliZ are expressed from a single operon, and are activated independently by FlhD_4_C_2_ and FliA (Kutsukake et al., 1999; Brown et al., 2008). Similarly, FliT and FliD are part of an operon and are also activated independently by FlhD_4_C_2_ and FliA (Kutsukake and Ide, 1995; Yokoseki et al., 1995).

FlgM binds with FliA to form the FlgM-FliA complex (Ohnishi et al., 1992; Chadsey and Hughes, 2001; Aldridge et al., 2006). Upon completion of the HBB structure, the FliA-FlgM interacts with the export apparatus leading to export/secretion of FlgM (Hughes et al., 1993; Aldridge et al., 2006). The newly freed FliA then activates class 3 genes (Gillen and Hughes, 1991a; Hughes et al., 1993; Chadsey et al., 1998; Chadsey and Hughes, 2001; Aldridge et al., 2006). FliD binds with FliT to form a FliD-FliT complex (Aldridge et al., 2010). This complex, upon interaction with the export apparatus, on completion of the HBB structure, exports FliD (Aldridge et al., 2010). The newly freed FliT forms a complex with FlhD_4_C_2_ and prevents activation of class 2 promoters (Yamamoto and Kutsukake, 2006; Aldridge et al., 2010). The P*ydiV* promoter is activated by the external stimulus of starvation and is repressed in rich media (Wada et al., 2011a). The YdiV protein enhances the rate of degradation of the FlhD_4_C_2_ complex and thereby acts as a negative regulator of class 2 gene expression (Wada et al., 2011b). FliZ represses expression from the P*ydiV* promoter and hence, aids class 2 gene expression via FlhD_4_C_2_ (Wada et al., 2011b).

In development of the model, the following assumptions were made.

- The regulatory network was modeled as a network of chemical reactor according to the scheme proposed by Gillespie (Gillespie, 1976, 1977).
- There is little evidence regarding the dynamics of assembly of multiple HBBs in a cell. In our model, we assume that HBBs are synthesized and subsequently assembled one after another in a sequential manner.
- Degradation rate of a protein was chosen to be either “high” or “low.” *In-vivo*, components such as FlhD_4_C_2_, HBB, FliT, FliZ, YdiV, and Class 3 genes are comparatively stable and thus degradation rates of these proteins/protein complex were considered to be low (Table 1). On the other hand, the degradation rates of FliA, FlgM, and FliD were considered to be comparatively higher (Table 1) as, free FliA,
  FlgM and FliD have been experimentally reported to be unstable (Fraser et al., 1999; Aldridge et al., 2006). Division of all variables in these two categories (with respect to protein degradation rate) helped reduce the number of free parameters used in the study considerably.
- At the start of each simulation (unless otherwise stated), we assume that the number of protein molecules of each species is zero.
- The rate of FlhD_4_C_2_ protein formation is assumed to be constant. The class 1 promoter is known to be regulated by a number of global regulators which feed in information regarding the environment and cellular physiology into the promoter (Ko and Park, 2000; Clarke and Sperandio, 2005). However, little is known about the mechanistic details and physiological significance of these individual interactions. Also, the operon encoding FlhD and FlhC has been reported to contain as much as six promoters (Yanagihara et al., 1999), indicating its complex regulation. In fact, in order to avoid the complexity associated with the class 1 promoter, many experimental flagellar studies work with a strain where the class 1 promoter is replaced with a *tetRA* cassette, making expression of flagellar genes, and their subsequent assembly contingent on addition of tetracycline to the media (Chilcott and Hughes, 2000; Saini et al., 2010a).
- In our model, we do not incorporate the roles of accessory regulators FliS and FlgN. FliS is the chaperone for the flagellin protein, FliC; and therefore, is implicated in controlling the filament length of the flagella (Auvray et al., 2001). Although, earlier studies have reported control of flagella number by FliS, we think that the observed phenomena may be the result of a polar mutation on *fliT* in that strain. FlgN is known to stabilize the HAPs FlgK and FlgL (Fraser et al., 1999). The HAP proteins are known to be required in small numbers for forming the hook-filament junction. Thus, we do not include this interaction in our model. While these two regulatory interactions could be incorporated in the model without making the model qualitatively complex, doing so would make the model increasingly cumbersome. Additionally, experimental studies reported on these interactions are relatively less, and hence, kinetic parameters associated with these interactions are much more open to speculation compared to the other regulatory interactions dictating flagellar biosynthesis.
- The model (for cell division) assumed linear growth of volume with time. As the cell volume becomes equal to or more than V_0_, the cell divides into two progenies, and the resultant protein molecules are split equally between the two resultant cells. Mathematically, for variable cell volume, the concentration of a species was replaced by the protein molecules divided by the cell volume at a given time.

**Table 1:** Details on the parameters used while building the model.

**Variable**	**Description**	**Value (in N h^−1^)**
K_1_	Class 1 promoter activity	3
K_2_	Activation coefficient for FlhDC dependent expression of HBB	5
K_2m_	Michaelis-Menten constant for FlhDC dependent expression of HBB	1,000
K_3_	Activation coefficient for FlhDC dependent expression of FlgM	50
K_3m_	Michaelis-Menten constant for FlhDC dependent expression of FlgM	2
K_4_	Activation coefficient for FlhDC dependent expression of FliAZ	2
K_4m_	Michaelis-Menten constant for FlhDC dependent expression of FliAZ	10
K_5_	Rate of FlgM secretion	2
K_6_	Activation coefficient for FliA dependent expression of class 3 genes	10
K_6m_	Michaelis-Menten constant for FliA dependent expression of class 3 genes	100
K_7_	Activation coefficient for FliA dependent expression of FlgM	10
K_7m_	Michaelis-Menten constant for FliA dependent expression of FlgM	1
K_8_	Activation coefficient for FliA dependent expression of FliAZ	10
K_8m_	Michaelis-Menten constant for FliA dependent expression of FliAZ	5
K_9_	Activation coefficient for FlhDC dependent expression of FliDT	1
K_9m_	Michaelis-Menten constant for FlhDC dependent expression of FliDT	1
K_10_	Activation coefficient for FliA dependent expression of FliDT	1
K_10m_	Michaelis-Menten constant for FliA dependent expression of FliDT	1
K_14_	Rate of FliD secretion	3
K_16_	Activation coefficient for FliZ dependent repression of YdiV	20
K_16m_	Michaelis-Menten constant for FliZ dependent repression of YdiV	1
k′	Constant for YdiV dependent repression of FlhDC	0.1
k_d_	Rate of degradation for FlhDC, HBB, FliT, FliZ, YdiV and Class 3 genes	0.1
k_d2_	Rate of degradation for FliA, FlgM and FliD	1
K_DCT_	Rate constant for FlhDC.FliT complex formation	0.8
K_DCTrev_	Rate constant for FlhDC.FliT complex disassociation	0.0004
K_AM_	Rate constant for FliA.FlgM complex formation	1,000
K_AMrev_	Rate constant for FliA.FlgM complex disassociation	0.3
K_DT_	Rate constant for FliD.FliT complex formation	6,500
K_DTrev_	Rate constant for FliD.FliT complex disassociation	6
α	Starvation level of the cell	[0–1]

*The parameters have been provided in terms of dimensionless concentration units. The disassociation rate of FliA-FlgM has been acquired from Chadsey et al. (1998)*.

The above described network (Figure 1) can be mathematically represented by the following ordinary differential equations:

(1) $$\begin{array}{l} \frac{\text{d}\left[\text{FlhDC}\right]}{\text{dt}}={\text{K}}_{1}-{\text{K}}_{\text{DCT}}\left[\text{FlhDC}\right]\left[\text{FliT}\right]\\ \;+\;K_{DCTrev}\left[FlhDC.FliT\right]\\ \;-\;{\text{k}}_{\text{d}}\left[\text{FlhDC}\right]\left(1+\text{k}\prime \left[\text{YdiV}\right]\right) \end{array}$$

(2) $$\begin{array}{l} \frac{\text{d}[\text{HBB}]}{\text{dt}}\;=\;\frac{{\text{K}}_{2}[\text{FlhDC}]}{{\text{K}}_{\text{2m}}+[\text{FlhDC}]}-{\text{k}}_{\text{d}}\left[\text{HBB}\right] \end{array}$$

(3) $$\begin{array}{l} \frac{\text{d}\left[\text{FliA}\right]}{\text{dt}}\;=\;\frac{{\text{K}}_{4}\left[\text{FlhDC}\right]}{{\text{K}}_{4\text{m}}+\left[\text{FlhDC}\right]}+\frac{{\text{K}}_{8}\left[\text{FliA}\right]}{{\text{K}}_{8\text{m}}+\left[\text{FliA}\right]}\\ -\;K_{AM}\left[FliA\right]\left[FlgM\right]\\ +\;{\text{K}}_{\text{AMrev}}\left[\text{FliA}.\text{FlgM}\right]-{\text{k}}_{\text{d2}}\left[\text{FliA}\right]\; \end{array}$$

(4) $$\begin{array}{l} \frac{\text{d}\left[\text{FlgM}\right]}{\text{dt}}=\frac{{\text{K}}_{3}\left[\text{FlhDC}\right]}{{\text{K}}_{3\text{m}}+\left[\text{FlhDC}\right]}+\frac{{\text{K}}_{7}\left[\text{FliA}\right]}{{\text{K}}_{7\text{m}}+\left[\text{FliA}\right]}\\ -{\text{K}}_{\text{AM}}\left[\text{FliA}\right]\left[\text{FlgM}\right]+{\text{K}}_{\text{AMrev}}\left[\text{FliA}.\text{FlgM}\right]\\ -\left[\text{HBB}\right]\left[\text{FliA}.\text{FlgM}\right]\;-{\text{k}}_{\text{d2}}\left[\text{FlgM}\right]\; \end{array}$$

(5) $$\begin{array}{l} \frac{\text{d}[\text{FliZ}]}{\text{dt}}=\;\frac{{\text{K}}_{4}[\text{FlhDC}]}{{\text{K}}_{\text{4m}}+[\text{FlhDC}]}+\frac{{\text{K}}_{8}[\text{FliA}]}{{\text{K}}_{8\text{m}}+[\text{FliA}]}-{\text{k}}_{\text{d}}\left[\text{FliZ}\right]\; \end{array}$$

(6) $$\begin{array}{l} \frac{\text{d}\left[\text{FliT}\right]}{\text{dt}}=\;\frac{{\text{K}}_{9}\left[\text{FlhDC}\right]}{{\text{K}}_{9\text{m}}+\left[\text{FlhDC}\right]}+\frac{{\text{K}}_{10}\left[\text{FliA}\right]}{{\text{K}}_{10\text{m}}++\left[\text{FliA}\right]}\\ -{\text{K}}_{\text{DT}}\left[\text{FliD}\right]\left[\text{FliT}\right]\\ +K_{DTrev}\left[FliD.FliT\right]-k_{d}\left[FliT\right]\; \end{array}$$

(7) $$\begin{array}{l} \frac{\text{d}\left[\text{FliD}\right]}{\text{dt}}=\;\frac{{\text{K}}_{9}\left[\text{FlhDC}\right]}{{\text{K}}_{9\text{m}}+\left[\text{FlhDC}\right]}+\frac{{\text{K}}_{10}\left[\text{FliA}\right]}{{\text{K}}_{10\text{m}}++\left[\text{FliA}\right]}\\ -{\text{K}}_{\text{DT}}\left[\text{FliD}\right]\left[\text{FliT}\right]+{\text{K}}_{\text{DTrev}}\left[\text{FliD}.\text{FliT}\right]\;\\ -\;{\text{K}}_{14}\left[\text{HBB}\right]\left[\text{FliD}.\text{FliT}\right]-\;{\text{k}}_{\text{d2}}\left[\text{FliD}\right]\; \end{array}$$

(8) $$\begin{array}{l} \frac{\text{d}[\text{YdiV}]}{\text{dt}}=\;\frac{{\text{K}}_{16}^{*}\alpha }{{\text{K}}_{16\text{m}}+[\text{FliZ}]}-\;{\text{k}}_{\text{d}}\left[\text{YdiV}\right]\; \end{array}$$

(9) $$\begin{array}{l} \frac{\text{d}[\text{Class}3]}{\text{dt}}=\;\frac{{\text{K}}_{6}[\text{FliA}]}{{\text{K}}_{6\text{m}}+[\text{FliA}]}-\;{\text{k}}_{\text{d}}\left[\text{Class}3\right] \end{array}$$

### Simulation using gillespie algorithm

The model was simulated using the Gillespie algorithm (Gillespie, 1976, 1977). The proteins from nine different genes in the model take part in 24 different reactions. Each of these reactions is represented by a propensity function. These propensity functions are taken from the rates of the corresponding reactions from the deterministic model. It is assumed that only one of these reactions happen at a particular time step and its probability of occurring is proportional to magnitude of its propensity function. This model was simulated for 10,000 cells and the average of these cells is reported.

Mutant cells here represent cells devoid of one or more genes from the above described network. Mutants are generated in the simulation by fixing the protein numbers corresponding to the mutation equal to zero. The activity of the P*flhDC* promoter represented class 1 activity. All proteins constituting structural parts of an HBB were assumed to be expressed from a single promoter. The activity of this promoter was taken to be representative class 2 promoter activity.

The HBB distribution data and figures represent the distribution of HBB expression among the 10,000 cells in the simulations. The distribution of HBB numbers in each cell once the steady state of expression was reached in the simulations is reported.

The cell division data and figures represent the expression of class 1, 2, and 3 genes after cell division. Here, it is assumed that the number of molecules of a particular protein is equally distributed between the two daughter cells. To generate this data, for a particular genotype (wild type or mutant) two sets of simulations are performed. The first simulation is run with initial values of all proteins as zero and the steady state expression levels of all the nine species are noted. These values are halved and are then used as initial value for the second simulation representing a daughter cell. The data is also normalized with the maximum expression value of the particular class of gene from the mother cell.

#### Sensitivity analysis

The sensitivity of parameters corresponding to four crucial reactions of the model: (1) The FliA-FlgM complex formation, (2) The FliT-FliD complex formation, (3) The FliT feedback i.e., interaction between FlhD_4_C_2_ and FliT, and (4) Activation of FlhD_4_C_2_ by FliZ (via YdiV), were analyzed. In each case the parameters describing the strength of association or disassociation are increased and decreased by 10% to see the effect on expression kinetics, HBB distribution at steady state, and kinetics after cell division.

#### Secretion analysis

The secretion analysis was performed to study the variation in expressions of class 2 and 3 genes with excretion of FlgM and FliD proteins out of the cell in the process of flagella formation. New variables representing “newly freed FlgM” and “newly freed FliD” are created in the simulation for FlgM and FliD proteins which are generated by breaking of their complexes with FliA and FliT respectively. The class 2 and 3 expressions at a particular time are then plotted with the cumulative amount of FlgM or FliD excreted till that time.

#### Starvation analysis

The simulations are run with three different values of the starvation variable α−0.1, 0.5, and 1 to check its effect on class 2 and class 3 expressions.

#### Mutants

Mutants are strains in which a particular reaction is silenced. Four different mutants are simulated:

- Activation of FliT by FliA is silenced (but FliT is still being activated by FlhD_4_C_2_). In this case, the FliT and FliD genes are not treated as operonic and the FliA-dependent activation term is deleted only in the equation of FliT and not from that of FliD.
- Activation of FliZ by FliA is silenced. In this case also, FliZ and FliA are not treated as in an operon and the FliA-dependent activation term is deleted only from the FliZ equation. Here FlhD_4_C_2_ still activates FliZ and FliA still positively auto regulates itself.
- Activation of FliT by FlhD_4_C_2_ is silenced. Here only the FlhD_4_C_2_-dependent activation of FliT equation is deleted and FliA is still able to activate FliT.
- Formation of FlhD_4_C_2_-FliT complex i.e., FliT-dependent suppression of class 2 promoter activation by FlhD_4_C_2_ is silenced.

Through these simulations, we wanted to understand the precise effect/logic of several of the regulatory proteins in the flagellar regulatory cascade, which are under the control of both, class 2 and class 3 promoters.

## Results

### Dynamics of flagellar gene expression

A distinctive feature of flagellar assembly dynamics in *Salmonella* pertains to expression of class 2 and class 3 genes in a sequential manner. That is, initiation of class 3 gene expression occurs only after the assembly of a functional HBB (Kutsukake et al., 1990; Karlinsey et al., 2000a,b; Kalir et al., 2001). This delay in induction of expression from class 3 promoters has been attributed to the FliA-FlgM interaction (Hughes et al., 1993; Kutsukake, 1994; Chadsey and Hughes, 2001; Aldridge et al., 2006). The FliA-FlgM check-point ensures that resource expenditure on production of class 3 genes only starts if the cell has built a functional base structure. The dynamics associated with class 2 and class 3 genes, and their dependence on various regulatory proteins in the network has been well characterized in a number of experimental studies (Ohnishi et al., 1992; Liu and Matsumura, 1995; Yanagihara et al., 1999; Kalir et al., 2001; Prüss et al., 2001; Aldridge et al., 2006, 2010; Yamamoto and Kutsukake, 2006; Saini et al., 2008).

We start by validating our model against these previously observed experimental results, and then explore the following two aspects of the assembly process. First, in a given population, there exists a distribution associated with number of flagella assembled per cell (Kusumoto et al., 2008; Balaban and Hendrixson, 2011). We use our model to explore this question in detail, and identify the quantitative roles played by various regulatory interactions in dictating this distribution. Secondly, most studies regarding flagella assembly dynamics consider assembly as cells transit from a non-flagellated to a flagellated state. However, experimentally, it has been known that the P*flhDC* promoter in *Salmonella* is never in a totally OFF condition (Karlinsey et al., 2000a). Hence, the non-flagellated state is perhaps not a representative of physiology of the organism. Lastly, we seek our model to explain how the cell adjusts its gene expression dynamics post cell-division, when number of flagella (and hence channels for secretion) per cell and cellular volume are halved.

**Figure 2:**
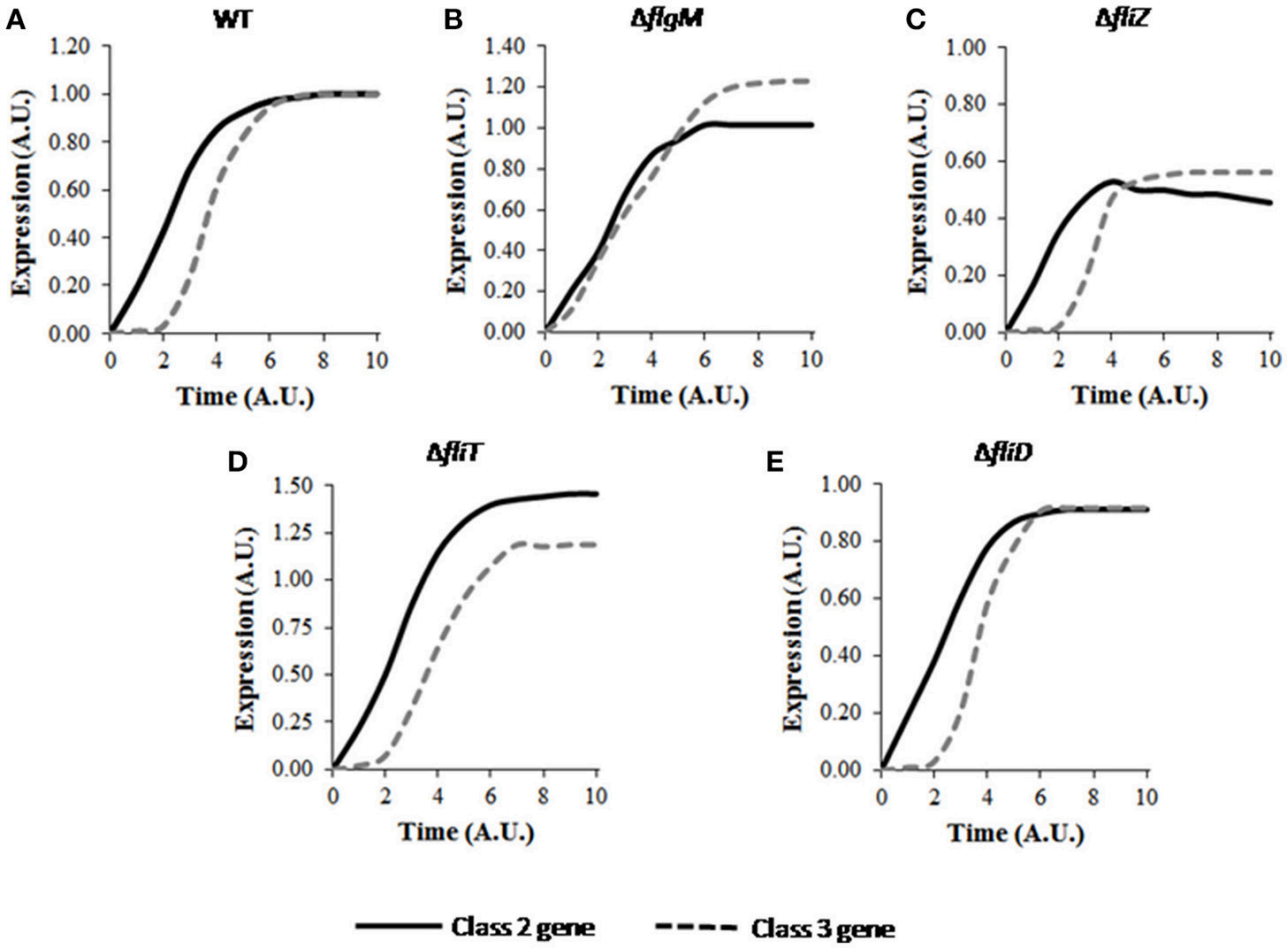
Dynamics of flagellar class 2 and class 3 gene expression in **(A)** wild type, **(B)** Δ*flgM*, **(C)** Δ*fliZ*, **(D)** Δ*fliT*, and **(E)** Δ*fliD*. Class 2 and class 3 genes have been represented by solid and dashed lines, respectively. Here, class 2 genes represent the genes encoding the constituent proteins of HBB. Class 3 genes include the genes encoding filament, motor and chemotaxis proteins. A. U. denotes arbitrary units. For mutants, the expression levels of class 2 and class 3 genes have been normalized with respect to those in wild type and subsequently plotted. The plot for wild type shows the characteristic delay in class 3 gene expression. FlgM is responsible for this delay as class 2 and class 3 genes are expressed at similar time in Δ*flgM* as shown in B. Deletion of the activators FliZ and FliD results in reduced expression levels of class 2 and class 3 genes, while, deletion of the repressor FliT leads to increase in the expression levels. Standard deviation in each curve is <10% of the mean values.

The model was first used to simulate the wild type expression dynamics of flagellar genes. The hierarchical expression of class 1, class 2, and class 3 genes (Figure 2) supports previous experimental observations (Karlinsey et al., 2000b; Saini et al., 2011). Further, simulations were performed for regulatory mutants and found to be in agreement with the previously reported studies. Simulation of the Δ*flgM* mutant resulted in initiation of class 2 and class 3 genes at the same time, eliminating the delay that was observed for class 3 gene in wild type and an enhanced expression of class 3 genes as compared to the wild type (Figure 2B; Brown et al., 2008; Saini et al., 2011). In the Δ*fliZ* mutant, expression levels of both class 2 and class 3 flagellar genes were shown to be reduced (Figure 2C; Saini et al., 2008). Simulation of the model with Δ*fliT* resulted in an increase in class 2 gene expression (Figure 2D), while a Δ*fliD* mutant exhibited reduced levels of both class 2 class 3, as compared to the wild type (Figure 2E). These results support previous experimental observations, and suggest that the model developed in the present study successfully captures the dynamics of flagellar gene expression in wild type and mutant strains.

### In an isogenic population, number of flagella assembled per cell varies as a normal distribution

The number of HBB structures assembled per cell has been suggested to be important for efficient movement (Schuhmacher et al., 2015). While too low a number is thought to leave the bacterium with too little flagella power for movement, building too many flagella are thought to mechanically interfere with each other and hinder the formation of an efficient bundle (Partridge and Harshey, 2013). An additional role for flagellar filaments during infection by *Salmonella* is its use to help the host launch an immune response (Hayashi et al., 2001). Since the quantum of the response launched by the host is likely dependent on the antigen amounts, the number of flagella control the immune response launched in the early stages of the infection. Hence, flagella number per cell is likely an important quantity both from the context of invasion leading to access to the host, and also, eliciting host immune response.

**Figure 3:**
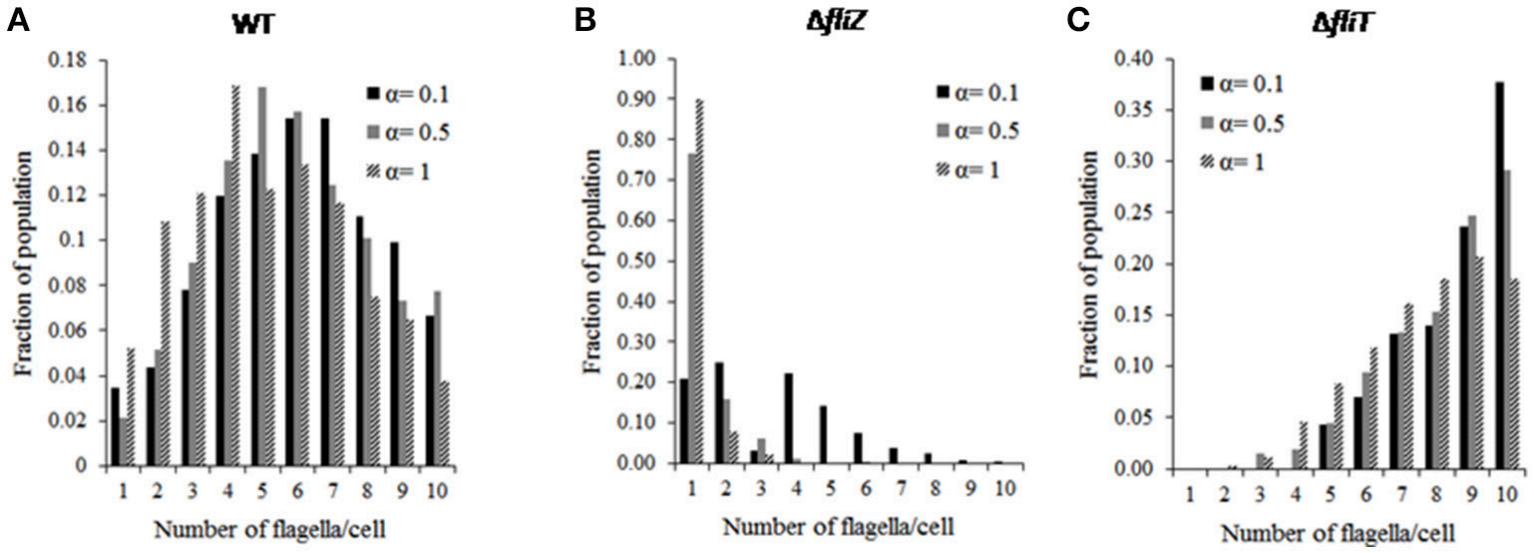
Distribution of flagella number (HBB number) in an isogenic population of **(A)** wild type, **(B)** Δ*fliZ*, **(C)** Δ*fliT* mutant cells at different starvation levels (represented by α). The HBB number follows a normal distribution in a wild type population. FliZ and FliT control HBB numbers in a nutritional status dependent manner. The influence of nutritional level on FliZ's regulatory effect is more than that of FliT.

Simulations were performed to study the cell-to-cell variability of an isogenic population with respect to HBB number. Our results show that, in wild type, number of flagella assembled per cell varies as a normal distribution (Figure 3A). This distribution qualitatively captures previously observed experimental results regarding distribution of number of flagella in an isogenic population (Saini et al., 2010a). Simulations were also performed with varying nutrition level, and the effect of the YdiV-dependent regulation was quantified. With increase in starvation level, the distribution moves leftwards, representing a lower number of HBBs per cell. More specifically, number of cells with lower HBB number was increased, with rising starvation level (Figure 3A). This result corroborates with earlier experimental studies where, under poor nutritional status, a large fraction of the cells in a population were observed to be devoid of any flagella (Aldridge et al., 2010). This trend is in contrast to *Salmonella*'s closely related species *E. coli*, where upon encountering starvation, the bacterium actively builds flagella—presumably to move to environments with greater availability of nutrients.

Next, our focus was to understand the effects of various mutations on the observed HBB distribution. Simulation of mutants indicated that, the mutations affect both the average and the standard deviation of the HBB numbers in a population. For example, the Δ*fliZ* mutant was observed to lead to a decrease in the number of flagella per cell (Figure 3B). In fact, in a Δ*fliZ* mutant, a significant fraction of cells were found to be harboring only single flagella. At high starvation level (starvation level 1), above 90% of total flagellated bacterial cells were observed to harbor only one flagella. This may indicate that, the effect of FliZ on population level distribution of flagella is more prominent at higher starvation level. Therefore, with increase in starvation level, the significance of FliZ in maintaining population level HBB distribution increases. Hence, the flagellar regulators FliT and FliZ are not only involved in controlling the dynamics of gene regulation, but actively control the flagella number per cell. In addition, the relative significance of the control loop associated with each regulator depends on the nutritional status of the cell.

Further, in a Δ*fliT* mutant, the distribution was observed to be skewed toward right at all starvation levels studied (Figure 3C). Thus, FliT is not only important for limiting over-expression of flagellar genes, but also helps maintain an optimal distribution of flagella numbers per cell in a population. This also suggests that the FliT-feedback loop acts in conjunction with the nutritional status of the cell to control flagellar numbers and their distribution. These results suggest an intricate link between flagellar biosynthesis and cellular metabolism, an aspect of flagellar gene regulation which has not been explored extensively.

### Dynamics of flagellar gene expression after cell division

The dynamics of flagellar gene expression post cell division, and the role played by the HBB structures in its control is not clearly understood. Post cell division, the flagella of a particular cell are distributed randomly between the two daughter cells. Thus, each daughter cell receives (roughly) half the number of flagella in the parent cell. In addition, changes in protein secretion rate (which is proportional to the number of HBBs in a cell) also affect the amount of proteins inside the cell, and likely change the dynamics of gene expression (and hence, flagella assembly) post division (Aizawa and Kubori, 1998). In order to understand the dynamics associated with this change (at cell division) and the associated adjustments, simulations of a model mimicking progeny cell were performed.

**Figure 4:**
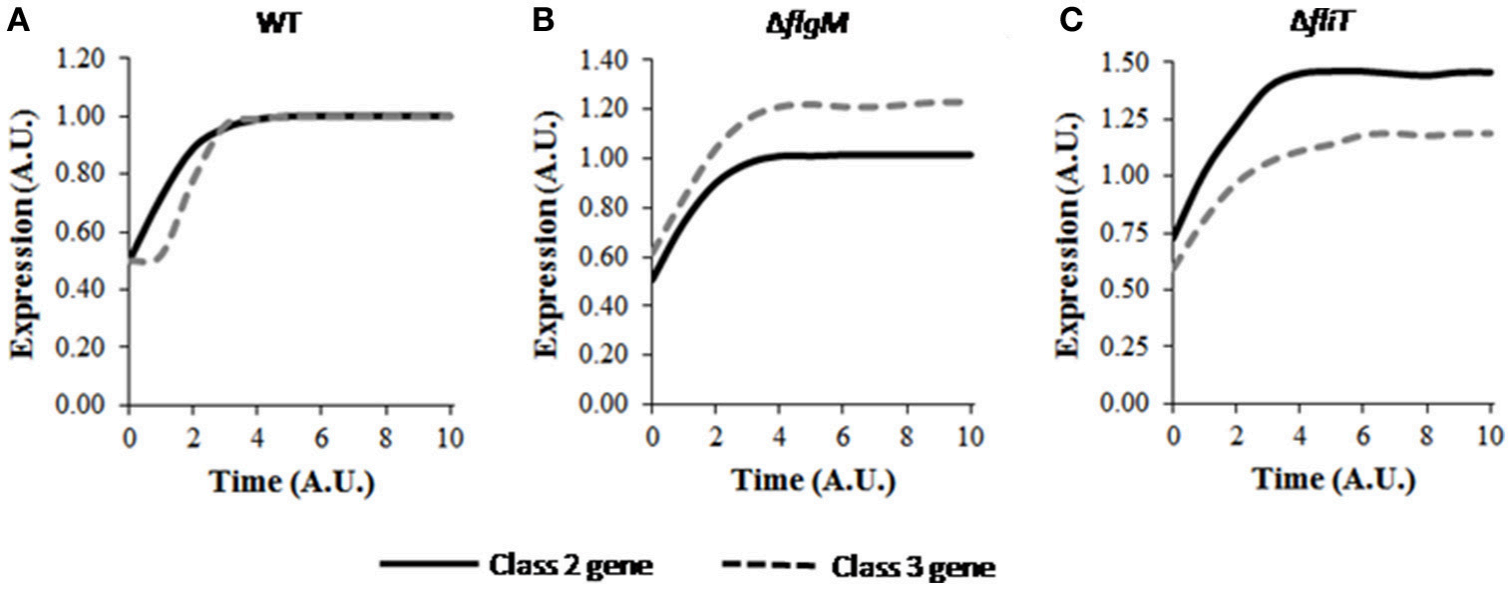
Dynamics of flagellar class 2 and class 3 gene expressions in **(A)** wild type, **(B)** Δ*flgM*, and **(C)** Δ*fliT* post cell division. Class 2 and class 3 genes have been represented by solid and dashed lines, respectively. A. U. denotes arbitrary units. For mutants, the expression levels of class 2 and class 3 genes have been normalized with respect to those in wild type and subsequently plotted. The delay in class 3 gene expression is also seen post cell division, similar to the observed phenomena during transition from non-flagellated to flagellated state. Δ*flgM* mutant shows altered hierarchy of class 2 and class 3 genes in a daughter cell, whereas, Δ*fliT* mutant leads to higher expression levels similar to its effect in a mother cell. Standard deviation in each curve is <10% of the mean values.

A wild type bacterium post division was observed to result in a delay in activation of class 3 genes with respect to that of class 2 genes (Figure 4A), similar to the behavior of a wild type (when studied in the context of transition from non-flagellated to a flagellated state; Kutsukake et al., 1990; Kalir et al., 2001). However, the post cell-division dynamics of class 3 genes was seen to be slower initially followed by a rapid increase in expression (Figure 4A) compared to wild type transitioning from non-flagellated to a flagellated state. As expression of excessive class 3 genes before sufficient HBB structures are assembled would presumably result in wastage of cellular resources, preservation of the expression hierarchy is critical for efficient assembly process. Additionally, premature expression of class 3 genes has been suggested to be harmful for the cell growth (Chilcott and Hughes, 2000). This is likely due to the fact that excessive expression of FliC in the cells leads to polymerization of the FliC protein intracellularly, which may affect cell viability. Thus, the current result suggests that, the gene expression dynamics is slightly altered in progeny in response cell division, while at the same time, the cell tries to maintain the hierarchy associated with class 2 and class 3 gene expression.

Further simulations were performed to study the effects of various mutants on the dynamics of flagellar genes while the cell adjusts to post cell division consequences. Altered hierarchy of class 2 and class 3 genes was also observed in the Δ*flgM* mutant (Figure 4B). Previous experimental studies have suggested the role of FlgM in preventing premature expression of class 3 genes by enforcing a time lag between expression initiation of class 2 and class 3 genes (Kutsukake et al., 1990; Karlinsey et al., 2000a,b; Kalir et al., 2001). The current result indicates that, FlgM is also important in maintaining the hierarchy immediately after cell division.

In the mutant Δ*fliT*, the steady state expression level of class 2 gene was observed to be around 1.5-fold higher than that of wild type (Figure 4C). This result may indicate that, similar to the role of FliT as repressor of class 2 gene expression in a mother cell (Yamamoto and Kutsukake, 2006), FliT-dependent feedback loop is required for maintaining level of class 2 gene. Further, increased levels of class 2 genes may be harmful for the cell as over-expression of flagella has been suggested to result in elevated host immune response, which in turn attenuates the pathogenesis (Yang et al., 2012). In addition, simulation of the Δ*fliT* mutant resulted in loss of the delay in expression of class 3 genes. This behavior of Δ*fliT* mutant was not apparent in a mother cell. Thus, it is possible that, post cell division, FliT plays a role in imposing the delay in class 3 gene expression, similar to the role of FlgM in a mother cell. On the other hand, the mutant Δ*fliZ* was observed to produce very low levels of both class 2 and class 3 genes. However, in this mutant, the hierarchy of gene expression was maintained post cell-division (data not shown).

### Roles of FliZ and FliT feedback loops on controlling flagellar gene expression at a population level and at a single-cell resolution

The regulatory network dictating flagella assembly includes feedback loops which help in timely and appropriate expression and assembly of flagellar genes. One such feedback loop corresponds to a positive feedback from FliZ that regulates expression of class 2 genes through a non-flagellar gene *ydiV* and the master regulator FlhD_4_C_2_ (Ohnishi et al., 1990). On the other hand, a feedback loop from FliT down-regulates class 2 gene expression in an FlhD_4_C_2_-dependent manner (Yamamoto and Kutsukake, 2006). Apart from these, two post-translational regulations through formation of protein complexes (FliA-FlgM and FliD-FliT) help in maintaining the temporal order and duration of flagellar gene expression (Aldridge et al., 2006, 2010; Saini et al., 2011). In order to better understand the role of these feedback loops in controlling the kinetics of flagellar genes, multiple simulations were performed with varying strength of the loops.

Simulation of a mutant progeny cell with 10-fold reduction in the strength of FliT feedback loop resulted in expression of class 2 and class 3 genes at almost same time (data not shown). Thus, similar to FlgM and FliZ (described in previous section), the role of FliT on expression hierarchy was not observed in normal growing condition, but was visible after cell division. Thus, it is possible that, FliT-dependent negative feedback loop accompanies FlgM and FliZ in preserving expression hierarchy of flagellar genes after the cell divides. Interestingly, simulation of the Δ*fliT* mutant didn't show any effect on hierarchical expression. This effect is likely significant only when other regulatory interactions involving FliT are functional. A mutant with 10-fold reduction in the association rate of FliA-FlgM complex also led to disruption of expression hierarchy. The role of FlgM in hierarchical activation of flagellar genes has been proposed by earlier studies (Kutsukake et al., 1990; Karlinsey et al., 2000a,b; Saini et al., 2011). The current model predicts that, even in presence of FlgM when the cell adapts to the changing environment after cell division, a certain critical minimum affinity for FliA-FlgM association is required for hierarchical expression of flagellar genes.

**Figure 5:**
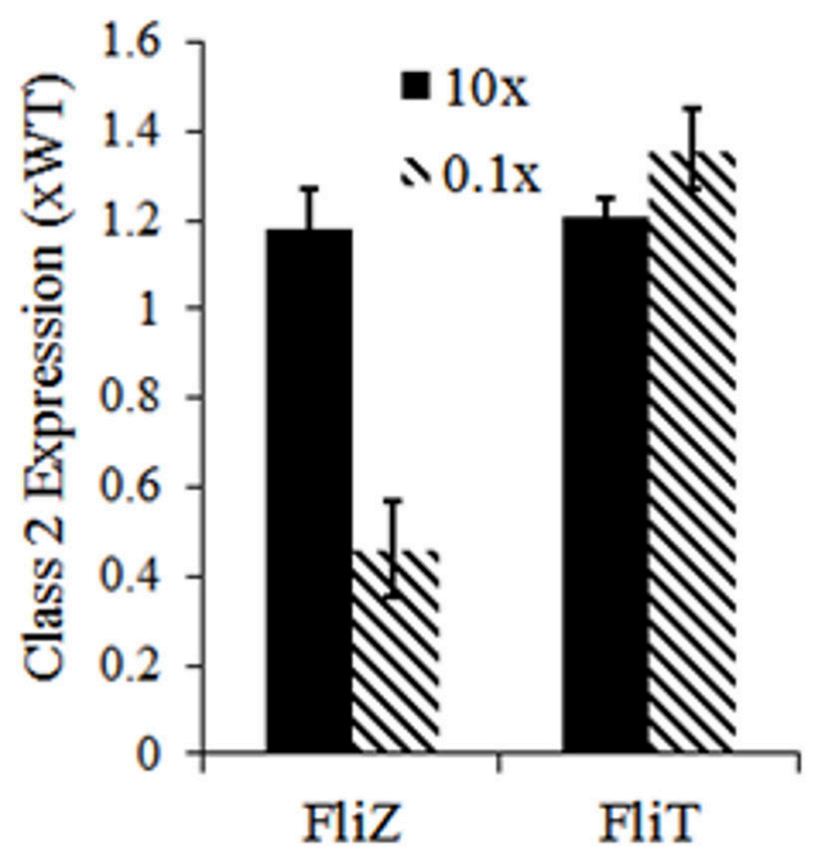
Effects of alterations in strength of FliZ and FliT feedback loops on class 2 gene expression. Here, class 2 gene expression represents the mean flagella number of a population of 10,000 cells. The values have been normalized to that of a wild type population. Reduction in the strengths of the feedback loops has a stronger influence on population level flagella number. Flagella number (and kinetics of flagellar gene expression) is sensitive to the strengths of the feedback loops.

Apart from cellular level dynamics, the current model predicts the roles of feedback loops in controlling population level dynamics of flagella gene expression. A 10-fold reduction in the association rate of FliD-FliT increased the number of cells producing lower number of flagella. Further simulations were performed with alterations in the parameters describing the strengths of FliZ and FliT feedback loops. The results suggest that, alterations in the strengths of the feedback loops, especially FliZ-feedback loop, affect flagella number per cell in a population (Figure 5). Hence, our model predicts that the feedback loops not only regulate flagella expression at cellular level, but also contribute significantly to the distribution of flagella number in an isogenic population, which in turn may govern the pathogenicity of *Salmonella*. These results also indicate that the feedback loops act under certain thresholds and any mutation resulting in alterations in the strengths of these loops can affect the kinetics of flagellar genes.

### FliZ and FliT regulate flagella in secretion dependent manner

Protein secretion plays an important role in flagellar assembly, and is thought to be used as a proxy for flagellar abundance in the cell (Brown et al., 2008; Saini et al., 2011). Secretion of FlgM from FliA-FlgM complex releases FliA inside the cell, which can then activate class 3 gene expression (Hughes et al., 1993; Kutsukake, 1994; Aldridge et al., 2006). FliT is a negative regulator of FlhD_4_C_2_ which acts once FliD, bound in FliD-FliT complex, is secreted from the cell (Aldridge et al., 2010). Thus, FliA-and FliT-dependent regulation of flagellar genes are controlled by rate of protein secretion. The FliZ-dependent positive feedback loop has been reported to be functional only at very low secretion rate or no secretion of FlgM (Saini et al., 2010a). In the current study, simulations were performed to understand dependence of various regulators on protein secretion.

**Figure 6:**
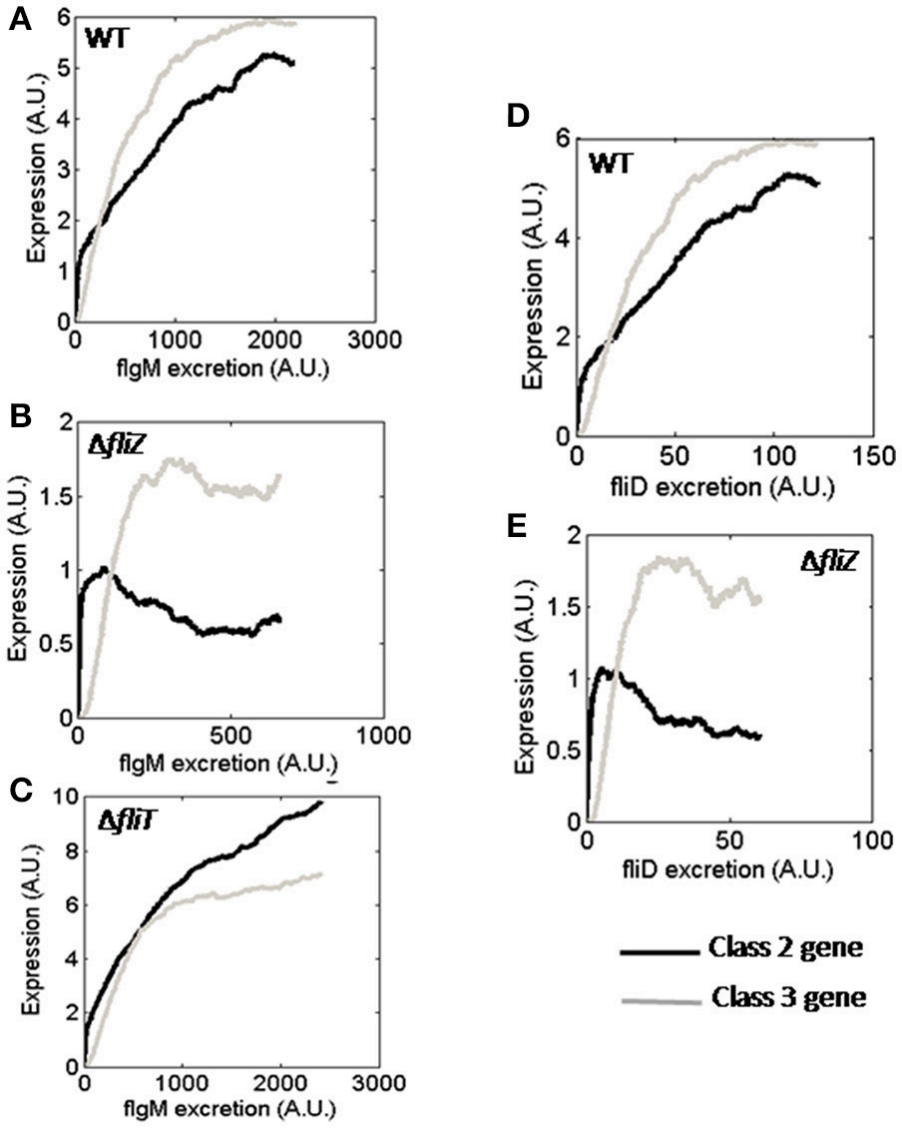
Class 2 and class 3 gene expression with varying FlgM secretion rate in **(A)** wild type, **(B)** Δ*fliZ*, and **(C)** Δ*fliT*. Class 2 and class 3 gene expression with varying FliD secretion rate in **(D)** wild type and **(E)** Δ*fliZ*. Class 2 and class 3 genes have been represented by Black and Gray solid lines, respectively. A. U. denotes arbitrary units.

In the simulated Δ*fliZ* mutant, the effect of FlgM secretion on the FliZ-dependent regulation of class 2 and class 3 genes could be observed (Figure 6B), as previously seen experimentally (Saini et al., 2010a). In addition, the behavior of Δ*fliT* mutant suggests that, the regulatory role played by FliT in expressions of class 2 and class 3 genes is probably more significant at higher secretion rate of FlgM (Figure 6C). Thus, the current results indicate that, not only FliA and FliZ, but FliT-dependent regulation of flagellar genes is also likely to be affected by FlgM secretion rate.

Next, expression of class 2 and class 3 genes with varying rates of FliD secretion were investigated. The result of Δ*fliZ* mutant indicates that, the regulatory effect of FliZ on class 2 and class 3 gene expressions is functional at even lower secretion rate of FliD than that of FlgM (Figure 6E). Thus, the current observations suggest that, the effects of flagellar regulators and protein secretion are correlated in a manner to facilitate optimal flagella assembly.

### Effect of starvation on class 3/class 2 ratio

**Figure 7:**
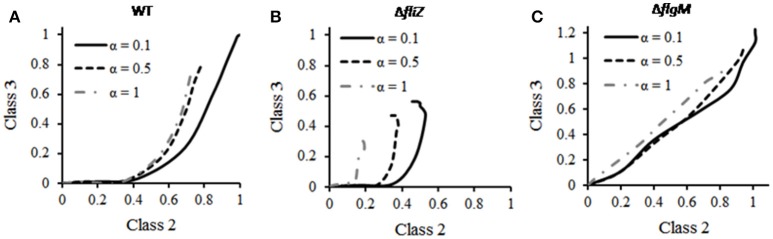
Effect of starvation on class 3/class2 gene ratio in **(A)** wild type, **(B)** Δ*fliZ*, and **(C)** Δ*flgM*. For mutants, the expression levels of class 2 and class 3 genes have been normalized with respect to those in wild type and subsequently plotted. In wild type, the ratio remains unaffected to changes in starvation level (α). Δ*fliZ* and Δ*flgM* mutants show altered pattern of this ratio. FliZ's role in maintaining the ratio is dependent on starvation level.

The relative abundance of class 3 and class 2 genes is likely critical for *Salmonella* in order to respond properly to the changes in environment. The effect of starvation on this ratio was investigated using the current model. Simulation of a wild type cell resulted in increase in the class 3/class 2 gene ratio with increasing class 2 gene expression (Figure 7). This indicates that, as class 2 genes (HBB) are produced, it indirectly accelerates expression of class 3 genes leading to increase in class3/class 2 ratio. This can be explained by earlier studies which reported that, completion of HBB leads to secretion of FlgM, which in turn increases the amount of free FliA inside the cell and this free FliA activates class 3 genes (Hughes et al., 1993; Chadsey et al., 1998; Chadsey and Hughes, 2001; Aldridge et al., 2006). In addition, the ratio was observed to be similar at three different starvation levels studied (0.1, 0.5, and 1; Figure 6). This suggests that, unlike the levels of flagellar genes, the class 3/class 2 gene ratio is robust to the nutritional status of the cell.

Further simulations were performed in order to analyze the effect of starvation on class 3/class 2 gene ratio in various mutants. The ratio in Δ*flgM* and Δ*fliZ* mutants was observed to differ from that of wild type. As shown in the Figure 6, the wild type exhibits a characteristic curve between the class 2 and class 3 trajectory with time. This trajectory is dependent on the starvation levels of the cell. With increasing starvation, the relative amounts of both, class 2 and 3 proteins decrease initially. However, beyond a certain value of starvation the levels of class 2 and class 3 proteins no longer drop further. However, this response to starvation conditions is not seen in the two mutants, as shown in Figure 6. In a Δ*fliZ* mutant, the expression levels of class 2 and class 3 proteins decrease continuously with increasing starvation conditions. While in a Δ*flgM* mutant, the expression levels of the proteins belonging to the two classes were seen to decrease with increasing starvation. However, in this case, the decrease was not as pronounced as in the Δ*fliZ* mutant. In addition, the dynamics of Δ*flgM* mutant are also characterized by the lack of delay in initiation of class 3 gene expression, upon induction of flagellar genes. Overall, our results show that, in response to starvation, the bacterium maintains an appropriate balance between class 2 and class 3 genes. This balance, however, is disturbed, when one of the two ancillary regulators (FliZ and FlgM) is removed from the network.

## Discussion

In the present study, a mathematical model has been used to understand two specific aspects of flagellar gene regulation in *Salmonella*. First, we quantify the role of various regulators in the network leading to cell-cell variation in the number of flagella assembled, and second, we study the impact of cell division in dictating dynamics of gene expression. Simulations of our models predict that the regulators FliZ and FliT not only control expression levels of flagellar genes at cellular level, but also play significant roles in maintaining optimal distribution of flagella in a population. The relative role of this effective control toward pathogenicity is unknown, although non-flagellated mutants are known to be not able to cause infection effectively in mouse models. In addition, our predictions suggest role of FlgM in controlling hierarchical expression of flagellar genes post cell-division. Our simulation results also indicate roles of FliZ- and FliT-dependent feedback loops in maintaining population level distribution of flagella. Further, we investigate the influence of FliZ and FlgM on class 3-class2 gene product ratio, a number likely critical for the pathogenicity of the bacterium. Overall, we propose that, the roles of the accessory regulators FliZ, FlgM, and FliT are far more complex than what is currently understood.

One of the limitations of the current model pertains to simplification of the regulatory effects governing activation of the genes encoding the master regulator of flagella, FlhD, and FlhC. The operon encoding FlhD and FlhC has been reported to be regulated by multiple regulators, many of which have not been identified yet (Kutsukake, 1997; Teplitski et al., 2003; Clarke and Sperandio, 2005). Thus, in the current study, activation of this operon has been represented by a step function representing the cumulative effect of all its regulators (Saini et al., 2011). Although, this seems to be a fair representation of actual behavior, availability of more experimental data in this regard may enhance the model efficiency. Another drawback corresponds to inability of the model to capture switching OFF of the flagella gene regulation. Earlier studies have suggested that, RtsB represses class 1 promoter, thus contributing to switching off the flagellar gene expression (Ellermeier and Slauch, 2003). Thus, two of the important aspects (of flagella) that remain unexplored are—(i) What are the primary factors that control switching OFF of the flagellar gene expression? (ii) How does the flagellar system interact with other virulence factors (like SPI1, SPI2, SPI4) in controlling expression dynamically and launching a successful infection (Bustamante et al., 2008; Saini et al., 2010b; Martínez et al., 2011)?

The flagellar machinery is a characteristic feature of many intestinal pathogens (apart from *S. typhimurium)*, such as *Escherichia coli, Pseudomonas aeruginosa, Clostridium difficile, Listeria monocytogenes* etc. (Hayashi et al., 2001; Bustamante et al., 2008; Saini et al., 2010a,b; Martínez et al., 2011; Yang et al., 2012). In addition to basic structural similarity, the flagella systems in all these bacteria have been suggested to participate in pathogenicity (Komeda, 1986; Shin and Park, 1995; Dasgupta et al., 2003; Gründling et al., 2004; Baban et al., 2013; Haiko and Westerlund-Wikström, 2013), resembling the physiological role it plays in *S. typhimurium*. In addition, the regulatory mechanism governing expression of flagella in *S. typhimurium* (involving the major regulators FlhD, FlhC, FliA, FliZ, FlgM, FliD, and FliT) has been found to be similar to that of *E. coli* (Chilcott and Hughes, 2000). For example, the master regulator, FlhD_4_C_2_, plays identical role in these two organisms (Chilcott and Hughes, 2000). However, the regulation of FlhD_4_C_2_ itself seems to differ. In *E. coli* OmpR has been shown to negatively regulate *flhDC* unlike in *S. typhimurium* (Shin and Park, 1995; Kutsukake, 1997). Further, the regulatory effect of FlgM (in coupling flagella assembly with late flagellar gene (class 3 gene) expression) observed in *S. typhimurium* has been reported to be also functional in *E. coli* (Komeda, 1986; Gillen and Hughes, 1991b). On the other hand, the regulatory mechanisms significantly differ in mono-flagellated bacteria like *P. Aeruginosa* (Dasgupta et al., 2003). In this organism, a four-tiered hierarchy of regulation has been shown to control flagella expression (Dasgupta et al., 2003). The regulation of flagella in *P. aeruginosa* was observed to involve a set of new genes (such as *fleQ, fleS, fleR* etc.) not found in either *S. typhimurium* or *E. coli*. Another such gene *mogR* has been reported to be an important repressor of flagella in *L. monocytogenes* where the overall regulatory mechanism has not been characterized in detail yet (Gründling et al., 2004). Apart from the above mentioned similarities and differences across various genera, serovar level variations have also been demonstrated. For example, even within *Salmonella*, the effect of flagellum on adhesion and invasion have been suggested to be serovar and/or host-target specific (Haiko and Westerlund-Wikström, 2013). Thus, it is likely that, the multi-factorial flagella machinery has evolved to be able to respond to diverse environment.

## Author contributions

CD, CM, SM, and SS: Conceptualized the work and the design of experiments; CD and CM: Carried out model construction, validation and simulation; CD, SM, and SS: Analyzed and interpreted the results; CD: Drafted the manuscript; SS and SM: Edited the manuscript; All authors have read and approved the manuscript.

### Conflict of interest statement

The authors declare that the research was conducted in the absence of any commercial or financial relationships that could be construed as a potential conflict of interest.

## References

[B1] Aizawa S. I. Kubori T. (1998). Bacterial flagellation and cell division. Genes Cells 3, 625–634. 10.1046/j.1365-2443.1998.00219.x 9893020

[B2] Aldridge C. Poonchareon K. Saini S. Ewen T. Soloyva A. Rao C. V.. (2010). The interaction dynamics of a negative feedback loop regulates flagellar number in *Salmonella enterica* serovar typhimurium. Mol. Microbiol. 78, 1416–1430. 10.1111/j.1365-2958.2010.07415.x 21143315

[B3] Aldridge P. D. Karlinsey J. E. Aldridge C. Birchall C. Thompson D. Yagasaki J.. (2006). The flagellar-specific transcription factor, sigma28, is the Type III secretion chaperone for the flagellar-specific anti-sigma28 factor FlgM. Genes Dev. 20, 2315–2326. 10.1101/gad.380406 16912280 PMC1553213

[B4] Auvray F. Thomas J. Fraser G. M. Hughes C. (2001). Flagellin polymerisation control by a cytosolic export chaperone. J. Mol. Biol. 308, 221–229. 10.1006/jmbi.2001.4597 11327763 PMC2528291

[B5] Baban S. T. Kuehne S. A. Barketi-Klai A. Cartman S. T. Kelly M. L. Hardie K. R.. (2013). The role of flagella in *Clostridium difficile* pathogenesis: comparison between a non-epidemic and an epidemic strain. PLoS ONE 8:e73026. 10.1371/journal.pone.0073026 24086268 PMC3781105

[B6] Balaban M. Hendrixson D. R. (2011). Polar flagellar biosynthesis and a regulator of flagellar number influence spatial parameters of cell division in *Campylobacter jejuni*. PLoS Pathog. 7:e1002420. 10.1371/journal.ppat.1002420 22144902 PMC3228812

[B7] Bennett J. C. Thomas J. Fraser G. M. Hughes C. (2001). Substrate complexes and domain organization of the Salmonella flagellar export chaperones FlgN and FliT. Mol. Microbiol. 39, 781–791. 10.1046/j.1365-2958.2001.02268.x 11169117 PMC2528293

[B8] Brown J. D. Saini S. Aldridge C. Herbert J. Rao C. V. Aldridge P. D. (2008). The rate of protein secretion dictates the temporal dynamics of flagellar gene expression. Mol. Microbiol. 70, 924–937. 10.1111/j.1365-2958.2008.06455.x 18811728

[B9] Bustamante V. H. Martínez L. C. Santana F. J. Knodler L. A. Steele-Mortimer O. Puente J. L. (2008). HilD-mediated transcriptional cross-talk between SPI-1 and SPI-2. Proc. Natl. Acad. Sci. U.S.A. 105, 14591–14596. 10.1073/pnas.0801205105 18799744 PMC2567235

[B10] Chadsey M. S. Hughes K. T. (2001). A multipartite interaction between Salmonella transcription factor sigma28 and its anti-sigma factor FlgM: implications for sigma28 holoenzyme destabilization through stepwise binding. J. Mol. Biol. 306, 915–929. 10.1006/jmbi.2001.4438 11237608

[B11] Chadsey M. S. Karlinsey J. E. Hughes K. T. (1998). The flagellar anti-sigma factor FlgM actively dissociates *Salmonella typhimurium* sigma28 RNA polymerase holoenzyme. Genes Dev. 12, 3123–3136. 10.1101/gad.12.19.3123 9765212 PMC317189

[B12] Chilcott G. S. Hughes K. T. (2000). Coupling of flagellar gene expression to flagellar assembly in *Salmonella enterica* serovar typhimurium and *Escherichia coli*. Microbiol. Mol. Biol. Rev. 64, 694–708. 10.1128/MMBR.64.4.694-708.2000 11104815 PMC99010

[B13] Clarke M. B. Sperandio V. (2005). Transcriptional regulation of flhDC by QseBC and sigma (FliA) in enterohaemorrhagic *Escherichia coli*. Mol. Microbiol. 57, 1734–1749. 10.1111/j.1365-2958.2005.04792.x 16135237

[B14] Clegg S. Hughes K. T. (2002). FimZ is a molecular link between sticking and swimming in *Salmonella enterica* serovar typhimurium. J. Bacteriol. 184, 1209–1213. 10.1128/jb.184.4.1209-1213.2002 11807085 PMC134799

[B15] Dasgupta N. Wolfgang M. C. Goodman A. L. Arora S. K. Jyot J. Lory S.. (2003). A four-tiered transcriptional regulatory circuit controls flagellar biogenesis in *Pseudomonas aeruginosa*. Mol. Microbiol. 50, 809–824. 10.1046/j.1365-2958.2003.03740.x 14617143

[B16] DiMarzio M. Shariat N. Kariyawasam S. Barrangou R. Dudley E. G. (2013). Antibiotic resistance in *Salmonella enterica* serovar typhimurium associates with CRISPR sequence type. Antimicrob. Agents Chemother. 57, 4282–4289. 10.1128/AAC.00913-13 23796925 PMC3754329

[B17] Ellermeier C. D. Slauch J. M. (2003). RtsA and RtsB coordinately regulate expression of the invasion and flagellar genes in *Salmonella enterica* serovar typhimurium. J. Bacteriol. 185, 5096–5108. 10.1128/JB.185.17.5096-5108.2003 12923082 PMC181000

[B18] Everest P. Ketley J. Hardy S. Douce G. Khan S. Shea J.. (1999). Evaluation of *Salmonella typhimurium* mutants in a model of experimental gastroenteritis. Infect. Immun. 67, 2815–2821. 10338486 10.1128/iai.67.6.2815-2821.1999 PMC96587

[B19] Francez-Charlot A. Laugel B. Van Gemert A. Dubarry N. Wiorowski F. Castanié-Cornet M. P.. (2003). RcsCDB His-Asp phosphorelay system negatively regulates the flhDC operon in *Escherichia coli*. Mol. Microbiol. 49, 823–832. 10.1046/j.1365-2958.2003.03601.x 12864862

[B20] Fraser G. M. Bennett J. C. Hughes C. (1999). Substrate-specific binding of hook-associated proteins by FlgN and FliT, putative chaperones for flagellum assembly. Mol. Microbiol. 32, 569–580. 10.1046/j.1365-2958.1999.01372.x 10320579

[B21] Galeva A. Moroz N. Yoon Y. H. Hughes K. T. Samatey F. A. Kostyukova A. S. (2014). Bacterial flagellin-specific chaperone FliS interacts with anti-sigma factor flgM. J. Bacteriol. 196, 1215–1221. 10.1128/JB.01278-13 24415724 PMC3957722

[B22] Gillen K. L. Hughes K. T. (1991a). Molecular characterization of flgM, a gene encoding a negative regulator of flagellin synthesis in *Salmonella typhimurium*. J. Bacteriol. 173, 6453–6459. 1655712 10.1128/jb.173.20.6453-6459.1991 PMC208980

[B23] Gillen K. L. Hughes K. T. (1991b). Negative regulatory loci coupling flagellin synthesis to flagellar assembly in *Salmonella typhimurium*. J. Bacteriol. 173, 2301–2310. 1848842 10.1128/jb.173.7.2301-2310.1991 PMC207783

[B24] Gillen K. L. Hughes K. T. (1993). Transcription from two promoters and autoregulation contribute to the control of expression of the *Salmonella typhimurium* flagellar regulatory gene flgM. J. Bacteriol. 175, 7006–7015. 10.1128/jb.175.21.7006-7015.1993 7693654 PMC206828

[B25] Gillespie D. T. (1976). A general method for numerically simulating the stochastic time evolution of coupled chemical reactions. J. Comp. Phys. 22, 403–434. 10.1016/0021-9991(76)90041-3

[B26] Gillespie D. T. (1977). Exact stochastic simulation of coupled chemical reactions. J. Phys. Chem. 81, 2340–2361. 10.1021/j100540a008

[B27] Gründling A. Burrack L. S. Bouwer H. G. A. Higgins D. E. (2004). *Listeria monocytogenes* regulates flagellar motility gene expression through MogR, a transcriptional repressor required for virulence. Proc. Natl. Acad. Sci. U.S.A. 101, 12318–12323. 10.1073/pnas.0404924101 15302931 PMC514476

[B28] Haiko J. Westerlund-Wikström B. (2013). The role of the bacterial flagellum in adhesion and virulence. Biology 2, 1242–1267. 10.3390/biology2041242 24833223 PMC4009794

[B29] Hayashi F. Smith K. D. Ozinsky A. Hawn T. R. Yi E. C. Goodlett D. R.. (2001). The innate immune response to bacterial flagellin is mediated by Toll-like receptor 5. Nature 410, 1099–1103. 10.1038/35074106 11323673

[B30] Hughes K. T. Gillen K. L. Semon M. J. Karlinsey J. E. (1993). Sensing structural intermediates in bacterial flagellar assembly by export of a negative regulator. Science 262, 1277–1280. 10.1126/science.8235660 8235660

[B31] Ikebe T. Iyoda S. Kutsukake K. (1999a). Promoter analysis of the class 2 flagellar operons of Salmonella. Genes Genet. Syst. 74, 179–183. 10650844 10.1266/ggs.74.179

[B32] Ikebe T. Iyoda S. Kutsukake K. (1999b). Structure and expression of the fliA operon of *Salmonella typhimurium*. Microbiology 145(Pt 6), 1389–1396. 10.1099/13500872-145-6-1389 10411266

[B33] Imada K. Minamino T. Kinoshita M. Furukawa Y. Namba K. (2010). Structural insight into the regulatory mechanisms of interactions of the flagellar type III chaperone FliT with its binding partners. Proc. Natl. Acad. Sci. U.S.A. 107, 8812–8817. 10.1073/pnas.1001866107 20421493 PMC2889304

[B34] Jain K. Pradhan A. Mokashi C. Saini S. (2015). Mathematical model of flagella gene expression dynamics in *Salmonella enterica* serovar typhimurium. Syst. Synth. Biol. 9, 19–31. 10.1007/s11693-015-9160-3 25972986 PMC4427578

[B35] Jones B. D. Lee C. A. Falkow S. (1992). Invasion by *Salmonella typhimurium* is affected by the direction of flagellar rotation. Infect. Immun. 60, 2475–2480. 1587617 10.1128/iai.60.6.2475-2480.1992 PMC257184

[B36] Jones C. J. Macnab R. M. (1990). Flagellar assembly in *Salmonella typhimurium*: analysis with temperature-sensitive mutants. J. Bacteriol. 172, 1327–1339. 10.1128/jb.172.3.1327-1339.1990 2407720 PMC208602

[B37] Kalir S. McClure J. Pabbaraju K. Southward C. Ronen M. Leibler S.. (2001). Ordering genes in a flagella pathway by analysis of expression kinetics from living bacteria. Science 292, 2080–2083. 10.1126/science.1058758 11408658

[B38] Karlinsey J. E. Lonner J. Brown K. L. Hughes K. T. (2000b). Translation/secretion coupling by type III secretion systems. Cell 102, 487–497. 10.1016/S0092-8674(00)00053-2 10966110

[B39] Karlinsey J. E. Tanaka S. Bettenworth V. Yamaguchi S. Boos W. Aizawa S. I.. (2000a). Completion of the hook-basal body complex of the *Salmonella typhimurium* flagellum is coupled to FlgM secretion and fliC transcription. Mol. Microbiol. 37, 1220–1231. 10.1046/j.1365-2958.2000.02081.x 10972838

[B40] Ko M. Park C. (2000). Two novel flagellar components and H-NS are involved in the motor function of *Escherichia coli*. J. Mol. Biol. 303, 371–382. 10.1006/jmbi.2000.4147 11031114

[B41] Komeda Y. (1986). Transcriptional control of flagellar genes in *Escherichia coli* K-12. J. Bacteriol. 168, 1315–1318. 10.1128/jb.168.3.1315-1318.1986 3536871 PMC213639

[B42] Kusumoto A. Shinohara A. Terashima H. Kojima S. Yakushi T. Homma M. (2008). Collaboration of FlhF and FlhG to regulate polar-flagella number and localization in Vibrio alginolyticus. Microbiology 154, 1390–1399. 10.1099/mic.0.2007/012641-0 18451048

[B43] Kutsukake K. (1994). Excretion of the anti-sigma factor through a flagellar substructure couples flagellar gene expression with flagellar assembly in *Salmonella typhimurium*. Mol. Gen. Genet. 243, 605–612. 8028576 10.1007/BF00279569

[B44] Kutsukake K. (1997). Autogenous and global control of the flagellar master operon, flhD, in *Salmonella typhimurium*. Mol. Gen. Genet. 254, 440–448. 10.1007/s004380050437 9180698

[B45] Kutsukake K. Ide N. (1995). Transcriptional analysis of the flgK and fliD operons of *Salmonella typhimurium* which encode flagellar hook-associated proteins. Mol. Gen. Genet. 247, 275–281. 10.1007/BF00293195 7770032

[B46] Kutsukake K. Ikebe T. Yamamoto S. (1999). Two novel regulatory genes, fliT and fliZ, in the flagellar regulon of Salmonella. Genes Genet. Syst. 74, 287–292. 10.1266/ggs.74.287 10791024

[B47] Kutsukake K. Ohya Y. Iino T. (1990). Transcriptional analysis of the flagellar regulon of *Salmonella typhimurium*. J. Bacteriol. 172, 741–747. 10.1128/jb.172.2.741-747.1990 2404955 PMC208501

[B48] Lehnen D. Blumer C. Polen T. Wackwitz B. Wendisch V. F. Unden G. (2002). LrhA as a new transcriptional key regulator of flagella, motility and chemotaxis genes in *Escherichia coli*. Mol. Microbiol. 45, 521–532. 10.1046/j.1365-2958.2002.03032.x 12123461

[B49] Liu X. Fujita N. Ishihama A. Matsumura P. (1995). The C-terminal region of the α subunit of *Escherichia coli* RNA polymerase is required for transcriptional activation of the flagellar level II operons by the FlhD/FlhC complex. J. Bacteriol. 177, 5186–5188. 10.1128/jb.177.17.5186-5188.1995 7665504 PMC177305

[B50] Liu X. Matsumura P. (1994). The FlhD/FlhC complex, a transcriptional activator of the *Escherichia coli* flagellar class II operons. J. Bacteriol. 176, 7345–7351. 10.1128/jb.176.23.7345-7351.1994 7961507 PMC197124

[B51] Liu X. Matsumura P. (1995). An alternative sigma factor controls transcription of flagellar class-III operons in *Escherichia coli*: gene sequence, overproduction, purification and characterization. Gene 164, 81–84. 10.1016/0378-1119(95)00480-T 7590326

[B52] Macnab R. M. (1999). The bacterial flagellum: reversible rotary propellor and type III export apparatus. J. Bacteriol. 181, 7149–7153. 10572114 10.1128/jb.181.23.7149-7153.1999 PMC103673

[B53] Martínez L. C. Yakhnin H. Camacho M. I. Georgellis D. Babitzke P. Puente J. L.. (2011). Integration of a complex regulatory cascade involving the SirA/BarA and Csr global regulatory systems that controls expression of the Salmonella SPI-1 and SPI-2 virulence regulons through HilD. Mol. Microbiol. 80, 1637–1656. 10.1111/j.1365-2958.2011.07674.x 21518393 PMC3116662

[B54] Ohnishi K. Kutsukake K. Suzuki H. Iino T. (1990). Gene fliA encodes an alternative sigma factor specific for flagellar operons in *Salmonella typhimurium*. Mol. Gen. Genet. 221, 139–147. 10.1007/BF00261713 2196428

[B55] Ohnishi K. Kutsukake K. Suzuki H. Lino T. (1992). A novel transcriptional regulation mechanism in the flagellar regulon of *Salmonella typhimurium*: an antisigma factor inhibits the activity of the flagellum-specific sigma factor, sigma F. Mol. Microbiol. 6, 3149–3157. 10.1111/j.1365-2958.1992.tb01771.x 1453955

[B56] Partridge J. D. Harshey R. M. (2013). Swarming: flexible roaming plans. J. Bacteriol. 195, 909–918. 10.1128/JB.02063-12 23264580 PMC3571328

[B57] Prüss B. M. Liu X. Hendrickson W. Matsumura P. (2001). FlhD/FlhC-regulated promoters analyzed by gene array and lacZ gene fusions. FEMS Microbiol. Lett. 197, 91–97. 10.1016/S0378-1097(01)00092-1 11287152

[B58] Saini S. Brown J. D. Aldridge P. D. Rao C. V. (2008). FliZ is a posttranslational activator of FlhD4C2-dependent flagellar gene expression. J. Bacteriol. 190, 4979–4988. 10.1128/JB.01996-07 18469103 PMC2447003

[B59] Saini S. Floess E. Aldridge C. Brown J. Aldridge P. D. Rao C. V. (2011). Continuous control of flagellar gene expression by the σ28-FlgM regulatory circuit in *Salmonella enterica*. Mol. Microbiol. 79, 264–278. 10.1111/j.1365-2958.2010.07444.x 21166907

[B60] Saini S. Koirala S. Floess E. Mears P. J. Chemla Y. R. Golding I.. (2010a). FliZ induces a kinetic switch in flagellar gene expression. J. Bacteriol. 192, 6477–6481. 10.1128/JB.00751-10 20935096 PMC3008537

[B61] Saini S. Slauch J. M. Aldridge P. D. Rao C. V. (2010b). Role of cross talk in regulating the dynamic expression of the flagellar salmonella pathogenicity Island 1 and type 1 fimbrial genes. J. Bacteriol. 192, 5767–5777. 10.1128/JB.00624-10 20833811 PMC2953706

[B62] Schuhmacher J. S. Thormann K. M. Bange G. (2015). How bacteria maintain location and number of flagella? FEMS Microbiol. Rev. 39, 812–822. 10.1093/femsre/fuv034 26195616

[B63] Shin S. Park C. (1995). Modulation of flagellar expression in *Escherichia coli* by acetyl phosphate and the osmoregulator OmpR. J. Bacteriol. 177, 4696–4702. 10.1128/jb.177.16.4696-4702.1995 7642497 PMC177235

[B64] Sperandio V. Torres A. G. Kaper J. B. (2002). Quorum sensing *Escherichia coli* regulators B and C (QseBC): a novel two-component regulatory system involved in the regulation of flagella and motility by quorum sensing in *E*. coli. Mol. Microbiol. 43, 809–821. 10.1046/j.1365-2958.2002.02803.x 11929534

[B65] Takaya A. Erhardt M. Karata K. Winterberg K. Yamamoto T. Hughes K. T. (2012). YdiV: a dual function protein that targets FlhDC for ClpXP-dependent degradation by promoting release of DNA-bound FlhDC complex. Mol. Microbiol. 83, 1268–1284. 10.1111/j.1365-2958.2012.08007.x 22380597 PMC4265217

[B66] Teplitski M. Goodier R. I. Ahmer B. M. M. (2003). Pathways leading from BarA/SirA to motility and virulence gene expression in Salmonella. J. Bacteriol. 185, 7257–7265. 10.1128/JB.185.24.7257-7265.2003 14645287 PMC296259

[B67] Tomoyasu T. Ohkishi T. Ukyo Y. Tokumitsu A. Takaya A. Suzuki M.. (2002). The ClpXP ATP-dependent protease regulates flagellum synthesis in *Salmonella enterica* serovar typhimurium. J. Bacteriol. 184, 645–653. 10.1128/JB.184.3.645-653.2002 11790733 PMC139528

[B68] Wada T. Morizane T. Abo T. Tominaga A. Inoue-Tanaka K. Kutsukake K. (2011a). EAL domain protein YdiV acts as an anti-FlhD4C2 factor responsible for nutritional control of the flagellar regulon in *Salmonella enterica* serovar typhimurium. J. Bacteriol. 193, 1600–1611. 10.1128/JB.01494-10 21278297 PMC3067674

[B69] Wada T. Tanabe Y. Kutsukake K. (2011b). FliZ acts as a repressor of the ydiv gene, which encodes an anti-FlhD4C2 factor of the flagellar regulon in *Salmonella enterica* serovar typhimurium. J. Bacteriol. 193, 5191–5198. 10.1128/JB.05441-11 21804009 PMC3187388

[B70] Wei B. L. Brun-Zinkernagel A. M. Simecka J. W. Prüss B. M. Babitzke P. Romeo T. (2001). Positive regulation of motility and flhDC expression by the RNA-binding protein CsrA of *Escherichia coli*. Mol. Microbiol. 40, 245–256. 10.1046/j.1365-2958.2001.02380.x 11298291

[B71] Yamamoto S. Kutsukake K. (2006). FliT acts as an anti-FlhD2C2 factor in the transcriptional control of the flagellar regulon in *Salmonella enterica* serovar typhimurium. J. Bacteriol. 188, 6703–6708. 10.1128/JB.00799-06 16952964 PMC1595477

[B72] Yanagihara S. Iyoda S. Ohnishi K. Iino T. Kutsukake K. (1999). Structure and transcriptional control of the flagellar master operon of *Salmonella typhimurium*. Genes Genet. Syst. 74, 105–111. 10.1266/ggs.74.105 10586519

[B73] Yang X. Thornburg T. Suo Z. Jun S. Robison A. Li J.. (2012). Flagella overexpression attenuates salmonella pathogenesis. PLoS ONE 7:e46828. 10.1371/journal.pone.0046828 23056473 PMC3463563

[B74] Yokoseki T. Kutsukake K. Ohnishi K. Iino T. (1995). Functional analysis of the flagellar genes in the fliD operon of *Salmonella typhimurium*. Microbiology 141(Pt 7), 1715–1722. 10.1099/13500872-141-7-1715 7551038

[B75] Zhang S. Kingsley R. A. Santos R. L. Andrews-Polymenis H. Raffatellu M. Figueiredo J.. (2003). Molecular pathogenesis of *Salmonella enterica* serotype typhimurium-induced diarrhea. Infect. Immun. 71, 1–12 10.1128/IAI.71.1.1-12.2003 12496143 PMC143292

